# Renal Mass Found on Imaging of Spine for Back Pain: An Incidental Finding

**DOI:** 10.7759/cureus.31267

**Published:** 2022-11-08

**Authors:** Megan E Andrews, Guirlette Grodecki, Christian Belisario, Sharvari S Dalal, Yejia Zhang

**Affiliations:** 1 Physical Medicine and Rehabilitation, Hospital of the University of Pennsylvania, Philadelphia, USA; 2 Sections of Ambulatory Care and Women's Health, Corporal Michael J. Crescenz Veterans Affairs Medical Center, Philadelphia, USA; 3 Section of Pathology and Laboratory Medicine, Corporal Michael J. Crescenz Veterans Affairs Medical Center, Philadelphia, USA

**Keywords:** surgery, metanephric adenoma, histology, incidental finding, renal tumor, lumbar spine, back pain

## Abstract

A middle-aged female patient who presented with back pain was found incidentally to have a renal mass by magnetic resonance imaging (MRI). Further imaging, including computerized tomography (CT) with contrast, suggested a high likelihood of malignancy. Following surgical resection, the tumor was found to be a rare benign lesion on subsequent pathological examination. The patient had conservative treatment for her presenting spine issues and is doing very well. Prompt work-up and treatment of incidental findings by the team of primary care, physical medicine and rehabilitation physicians, radiologists, pathologists, and surgeons helped to ensure a good outcome. Residents had a learning opportunity about the disease and on timely management of incidental findings.

## Introduction

Physicians often encounter unanticipated findings unrelated to the original diagnostic inquiries (incidental findings). Increased use of advanced diagnostic modalities has increased the incidental diagnosis of asymptomatic entities.

The metanephric adenoma is a rare, benign, embryonal-epithelial neoplasm of the kidney. It is usually discovered incidentally with no presenting symptoms. Presenting symptoms such as hematuria, pyrexia, flank pain, abdominal mass, or weight loss indicate advanced disease. A metanephric adenoma is more common in females. Most of the tumors present as unilateral and unifocal, circumscribed but not encapsulated lesions [1].

A renal tumor with radiological features of malignancy was found on imaging for a patient with chronic back pain. After tumor removal, histological examination confirmed that it was a rare benign tumor. Proper management of these findings often requires teamwork and is key to ensuring good outcomes for patients and limiting malpractice liabilities for physicians.

## Case presentation

A 55-year-old woman with a past medical history of type II diabetes, obesity, tobacco use disorder, bilateral knee pain, and low back pain presented to the acupuncture clinic for pain management. She described a history of constant, aching pain in the low back with radiation into the left buttock and posterior thigh for 10 years, associated with numbness of both thighs. The pain was aggravated by flexion of the lumbar spine and alleviated by extension. Prior imaging indicated three herniated intervertebral discs and facet arthropathy throughout the lumbar spine. Interventions included physical therapy, lumbar selective nerve root blocks, and facet joint injections. Medications included gabapentin, cyclobenzaprine, nabumetone, and methylprednisolone. She was referred to a board-certified physiatrist who performs acupuncture.

Physical examination showed the strength of bilateral legs 5/5. The patellar tendon and heel tendon reflexes were 1+ and 1, respectively, with intact sensation to light touch. Straight leg raising test caused radiating pain to the buttock and posterior thigh bilaterally. 

**Figure 1:**
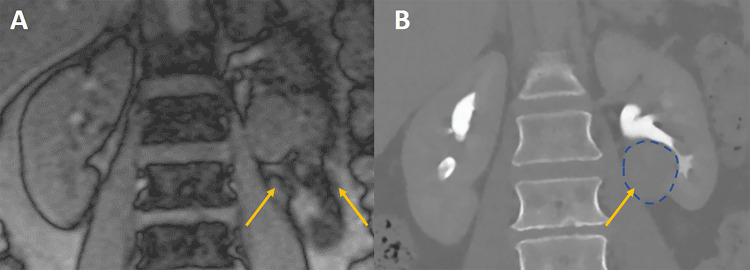
Coronal images of the thoracolumbar spine, capturing the kidneys A: magnetic resonance image (MRI); B: computerized tomography (CT) image. Arrows show a mass in the left kidney (outlined with a discontinuous superimposed circle).

Given her persistent back pain with radicular symptoms, repeat X-ray and MRI of the lumbar spine were ordered. X-ray showed grade I spondylolisthesis at lumbar (L) 4/5 with evidence for instability with flexion. MRI showed severe bilateral facet joint degeneration at L4/5 and L5/S1 and an L5/S1 herniated disc with superimposed right lateral disc extrusion. MRI of the lumbar spine also showed an incidental finding - a lesion approximately 3 cm in diameter in the lower pole of the left kidney (Figure 1A), consistent with a solid mass or complex solid-cystic lesion. The border between the renal mass and surrounding tissues appeared ill-defined on MRI, suggesting malignancy and/or local responses. CT with contrast revealed a partially exophytic heterogeneous solid renal mass (Figure 1B).

The patient was referred to surgery and underwent a robotic-assisted minimally invasive left partial nephrectomy. After surgery, the patient continued to take medication for her low back pain. Her spondylolisthesis, not her renal tumor, was considered the generator of her pain. The patient had physical therapy, and mild to moderate aerobic exercises were encouraged.

**Figure 2:**
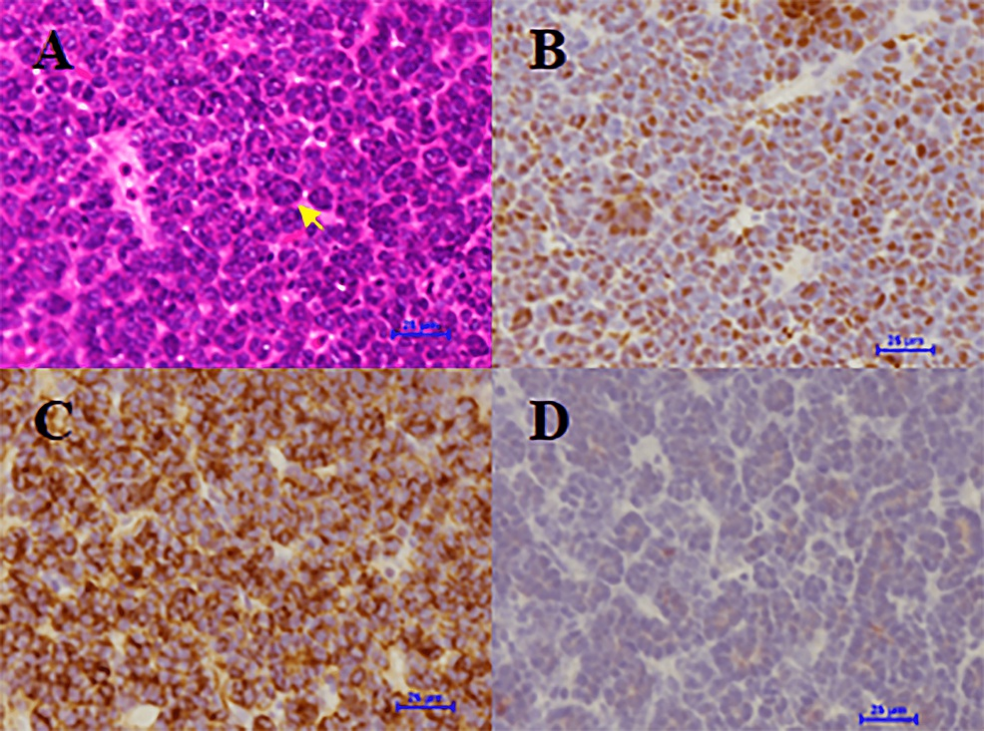
Histological features of the tumor characteristic of metanephric adenoma A: hematoxylin and eosin staining; arrow points to the tubule-like structure; B: immunostaining for Wilms tumor (WT)1; C: immunostaining for a cluster of differentiation (CD)57; D: immunostaining for cytokeratin (CK)7. Scale bars: 25 µm

Post-resection gross examination of the specimen revealed a tan-grey, soft to firm homogeneous, relatively well-circumscribed mass in the lower pole of the left kidney measuring 3.8x3.2x2.6 cm. The tissue was embedded in paraffin and prepared for histological examination. Hematoxylin and eosin (H&E) staining showed a cellular blue tumor in closely packed tubular structures with cytologically benign-looking tumor cells (Figure 2A). Immunohistochemical studies showed that the tumor was positive for Wilms tumor 1 (WT1) and a cluster of differentiation 57 (CD57) and negative for cytokeratin 7 (CK7; Figure 2B-D). This combination of markers is considered pathognomonic for metanephric adenoma [2-4]. 

## Discussion

Metanephric adenoma, which derives from the embryonic tissue of the nephritic medulla, is a very rare benign tumor accounting for about 0.2% of adult renal epithelial neoplasms and can mimic malignancy clinically and radiologically. The differential diagnosis in this age group includes a solid variant of papillary renal cell carcinoma, metastatic thyroid papillary carcinoma, and metanephric adenoma, and a number of other types [5-7]. Distinguishing these entities is crucial because the survival rate for patients with different types of tumors varies widely [8]; therefore, patient management needs to be based on the diagnosis.

Most grade I spondylolisthesis can be treated conservatively, without surgery [9,10]. This patient has continued to follow up with acupuncture for pain management biweekly for six months. She has benefited from an integrative whole health approach and has made many lifestyle changes. She has lost 10-15 pounds since learning of the renal mass and remains committed to a healthy lifestyle. With continued weight loss, she is expected to defer surgical intervention for her back pain. She has appreciated the collaborative, multi-disciplinary approach to her care and credits all teams for her positive outcome.

## Conclusions

A patient who presented with back pain was found incidentally to have a renal mass by imaging, which suggested a high likelihood of malignancy. Following surgical resection, the tumor was found to be a rare benign lesion. The patient had conservative treatment for her presenting spine issues and is doing very well. Prompt work-up and treatment of incidental findings by the team of physicians contributed to a good outcome. 
